# Early Drosophila Oogenesis: A Tale of Centriolar Asymmetry

**DOI:** 10.3390/cells10081997

**Published:** 2021-08-05

**Authors:** Maria Giovanna Riparbelli, Veronica Persico, Giuliano Callaini

**Affiliations:** 1 Department of Life Sciences, University of Siena, Via Aldo Moro 2, 53100 Siena, Italy; riparbelli@unisi.it (M.G.R.); persico@student.unisi.it (V.P.); 2 Department of Medical Biotechnologies, University of Siena, Via Aldo Moro 2, 53100 Siena, Italy

**Keywords:** Drosophila, oogenesis, oocyte development, centriole asymmetry

## Abstract

Among the morphological processes that characterize the early stages of Drosophila oogenesis, the dynamic of the centrioles deserves particular attention. We re-examined the architecture and the distribution of the centrioles within the germarium and early stages of the vitellarium. We found that most of the germ cell centrioles diverge from the canonical model and display notable variations in size. Moreover, duplication events were frequently observed within the germarium in the absence of DNA replication. Finally, we report the presence of an unusually long centriole that is first detected in the cystoblast and is always associated with the developing oocyte. This centriole is directly inherited after the asymmetric division of the germline stem cells and persists during the process of oocyte selection, thus already representing a marker for oocyte identification at the beginning of its formation and during the ensuing developmental stages.

## 1. Introduction

**Figure 1:**
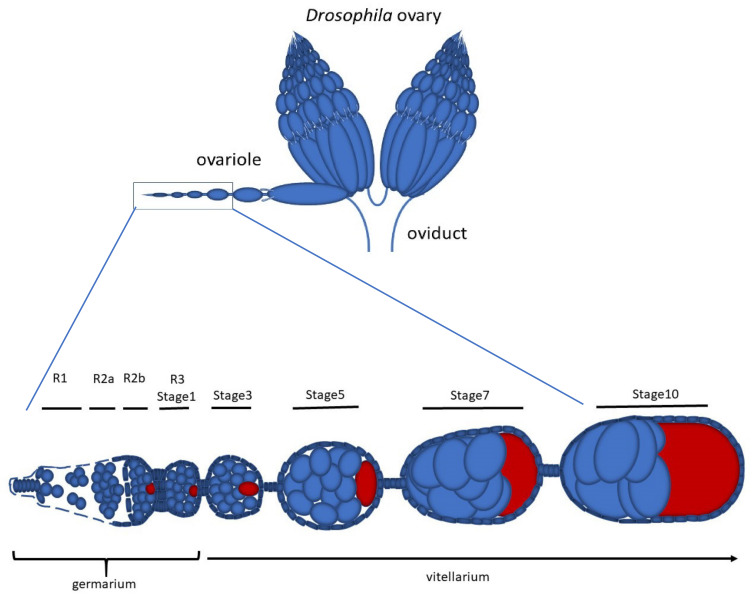
Schematic representation of the Drosophila ovary and detail of an ovariole showing stages of egg chamber development; the oocyte is red.

The Drosophila ovary consists of 16–18 ovarioles formed by a chain of egg chambers that mature progressively and move from the anterior to the distal region of the ovariole (Figure 1). Oogenesis begins in region 1 of the germarium, where two to three germline stem cells (GSCs) divide asymmetrically to originate a cystoblast and a new stem cell which remains attached to the somatic cap cells at the tip of the germarium. The cystoblast undergoes four rounds of incomplete mitotic divisions which, in region 2a of the germarium, lead to the formation of 16 cystocytes interconnected by cytoplasmic bridges, or ring canals [1]. In region 2a, all of the cells within one cyst look the same. The consecutive mitotic divisions result in the formation of cystocytes with different numbers of ring canals; the two older cells, the so-called pro-oocytes, have four ring canals each, whereas the other cells derived from the subsequent mitoses have one, two, or three ring canals depending on their age [2,3]. Only one of the two cystocytes with four ring canals will be the future oocyte. This cell is the former cystoblast and is directly derived from the first asymmetric division of the CSC.

The differentiation of the oocyte through the germarium can be followed by the accumulation of specific oocyte factors, the dynamic of the synaptonemal complex, the organization of the microtubule (MT) cytoskeleton, and the behaviour of the centrioles. Oocyte-specific proteins firstly accumulate in the cytoplasm of the two pro-oocytes, but from late stage 2a they are concentrated in the differentiating oocyte [4]. The synaptonemal complex that underlines the beginning of meiosis transiently appears during stage 2a in some cystocytes [5], but from late stage 2b it is restricted to the oocyte only [6]. The MT cytoskeleton is highly dynamic during early Drosophila oogenesis and undergoes a stereotypical spatial reorganization which correlates with oocyte specification and polarization [7,8,9]. MTs span the whole cytoplasm of the cystocytes in region 2a, but they polarize in region 2b with their minus ends restricted to one of the two pro-oocytes [6,7,9]. The MTs cross the ring canals and extend within the cytoplasm of the nurse cells [10], thus representing the preferential route for translocating the cytoplasmic components needed for oocyte growth and differentiation. Treatment with colchicine abolishes the differentiation of the oocyte and results in egg chambers containing 16 nurse cells [11].

Centriole clustering in only one cell of the cyst represents an additional and peculiar aspect that characterizes the selected oocyte [7,9,12]. Each of the 16 interconnected germline cells in region 2a display a centriole pair. In region 2b, however, one of the two cells with four ring canals accumulates more centrioles that travel from the nurse cells through the ring canals. Centrioles have been detected at the ultrastructural level in the oocyte cytoplasm during stage 9 [13], but typical centriole markers have been observed during later stages of oogenesis [14].

The mechanism that translocates the centriole from the nurse cells to the oocyte is unclear. Although the MT cytoskeleton seems to be dispensable for centriole accumulation to the oocyte [5,9], the journey of the centrioles from the nurse cells is sensitive to the MT-associated motor protein dynein [9]. This suggests the presence of drug-resistant MT bundles along which the centrioles can move. In support of this idea, it has been shown that antibodies against acetylated tubulin, a marker for stable microtubules, label a population of microtubules associated with the fusome [15].

All of the above markers allowed us to recognize the just-selected oocyte from stage 2a cysts, but they do not permit the identification of the oocyte at earlier stages. In contrast, during the asymmetric division of the GSCs, the cystoblast inherits one third of the spectrosome that gives origin to the fusome, a branched structure that expands during the following mitoses and links all the cells of the same cysts through the ring canals [16]. The fusome is asymmetrically distributed between the daughter cystocytes, and the former cystoblast maintains the older fusome material. The preferential accumulation of the fusome material to one cell is difficult to recognize during the four mitoses that lead to the formation of the 16 cystocytes, and the fusome soon disintegrates, becoming barely detectable.

Although the complex dynamics of the centrioles within the germarium were beautifully illustrated several years ago by transmission electron microscopy analysis [12], some aspects of their structural organization remain elusive and require additional attention. Therefore, we re-examined the first phases of Drosophila oogenesis and uncovered new, undescribed features that shed light on the centriole behaviour in the female germ cells.

## 2. Materials and Methods

### 2.1. Drosophila Strains

The Drosophila stock Oregon R was raised on a standard Drosophila medium in 200 mL plastic containers at 24 °C.

### 2.2. Transmission Electron Microscopy

Ovaries from adult flies were dissected in phosphate buffered saline (PBS) to isolate the anterior region of the ovarioles including the germarium and the early stages of the vitellarium. Next, the samples were fixed in 2.5% glutaraldehyde in PBS overnight at 4 °C. After rinsing for 20 min in PBS, the ovarioles were post-fixed in 1% osmium tetroxide in PBS for 1 h at 4 °C. The samples were then dehydrated through a graded series of ethanol infiltrated with a mixture of Epon–Araldite resin and polymerized at 60 °C for 48 h. Ultrathin sections were cut with a Reichert ultramicrotome, collected with formvar-coated copper grids, and stained with uranyl acetate and lead citrate. We used single slot grids with oval holes to make possible the observations of the serial sections. TEM preparations were observed with a Tecnai G2 Spirit EM (FEI) equipped with a Morada CCD camera (Olympus).

## 3. Results 

### 3.1. An Unusual Centriole in the Oocyte Cytoplasm

**Figure 2:**
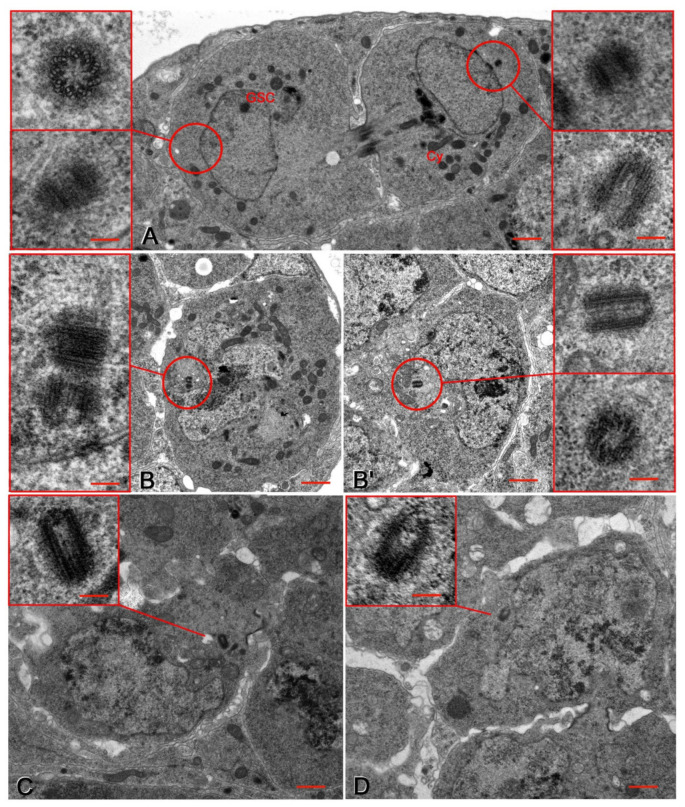
Region 1 (**A**,**B**,**B’**) and region 2a (**C**,**D**) of the Drosophila germarium. Circles in (**A**,**B**,**B’**) denote the pole regions where the serial sections uncover the centrioles at different focal planes that are not simultaneously visible in a single section. (**A**) Telophase of the asymmetric division of a germline stem cell (GSC); the spindle pole of the GSC has two short orthogonal centrioles, whereas the cystoblast (Cy) displays a short centriole and an elongated one (left and right insets). (**B**,**B’**) Cross sections of two interphase sister cells originated by the first division of the cystoblast; the cell in (**B’**) displays one short centriole and an elongated one, whereas the cell in (**B**) has two short centrioles (left and right insets). (**C**,**D**) Detail of region 2a showing cystocytes from different cysts with elongated centrioles (insets, **C**,**D**). Bars: **A**–**D**, 1 μm; insets, 150 nm.

Each Drosophila ovariole consists of an anterior germarium and a posterior vitellarium that contains progressive maturing egg chambers. The germarium can be subdivided morphologically into four distinct regions [1,17]. Region 1 contains the germ line stem cells (GSCs) that divide asymmetrically to give origin to one stem cell, close to the cap cells, and a daughter cystoblast away from the tip of the germarium (Figure 2A). Both of the sister cells inherited a centrosome with a pair of centrioles. Remarkably, the centrosome of the GSCs consisted of two orthogonal centrioles that did not exceed 250 nm in length (Figure 2A), whereas the centrosome of the cystoblast contained a long mother centriole (330 ± 12 nm) and a shorter daughter (230 ± 17 nm; Figure 2A).

In region 1, the cystoblast underwent four rounds of incomplete mitotic divisions which formed a cyst of 16 interconnected cystocytes in region 2a. The elongated centriole remained in one of the two sister cells after the first division of the cystoblast (Figure 2B,B’) and was observed in only one cell of the cyst during the following mitotic divisions. Serial sections of six region 2a-cysts from four germaria revealed that only one single germ cell in each cyst displayed an elongated centriole (Figure 2C,D). 

**Figure 3:**
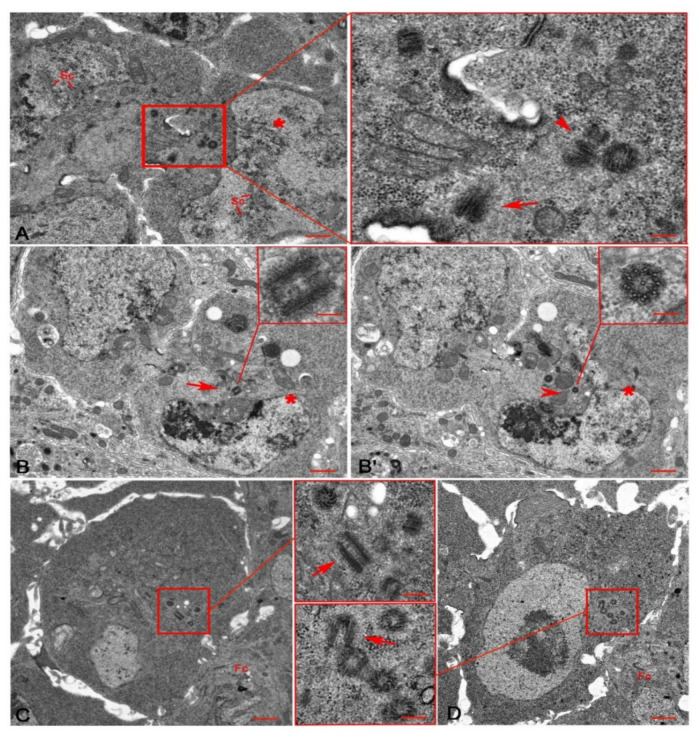
Region 2b (**A**,**B**,**B’**) of the germarium and stages 2 (**C**) and 3 (**D**) of the vitellarium. (**A**) Cross section showing two pro-oocytes with distinct synaptonemal complexes (Sc), the posterior one is the pro-oocyte (asterisk); magnification highlights two orthogonal parent centrioles (arrowhead) and the grazing section of a longer one (arrow) within the oocyte cytoplasm. (**B**,**B’**) Two consecutive serial sections of one oocyte (asterisks) showing an elongated centriole (arrow in **B**) and its daughter (arrowhead in **B’**). The daughter centriole consists of mixed singlet and doublet microtubules (inset, **B’**). Cross sections of two oocytes during stages 2 (**C**) and 3 (**D**); magnification (insets, **C**,**D**) shows centriole clusters in front of the follicle cells (Fc), arrows point to elongated centrioles. Bars: **A**–**D**, 1 μm; inset **A**, 150 nm; inset **B**,**B’**, 100 nm; insets **C**,**D**, 200 nm.

In region 2b, the two pro-oocytes maintained distinct synaptonemal complexes (Figure 3A) and the oocyte could be recognized in the posterior region of each cyst. Serial sections of fourteen region 2b-cysts showed five to nine short centrioles and one elongated centriole inside the cytoplasm of each oocyte (Figure 3A,B,B’).

During stage 2 (Figure 3C), when the germ cell cysts left the germarium and entered the vitellarium, and stage 3 (Figure 3D), the centrioles formed distinct clusters behind the oocyte nucleus and in front of the follicular epithelium. An elongated centriole remained present within these clusters (Figure 3C,D). The elongated centrioles were no longer detected in serial sections of stage 5 oocytes (not shown).

### 3.2. Centriole Duplication during Early Oogenesis

**Figure 4:**
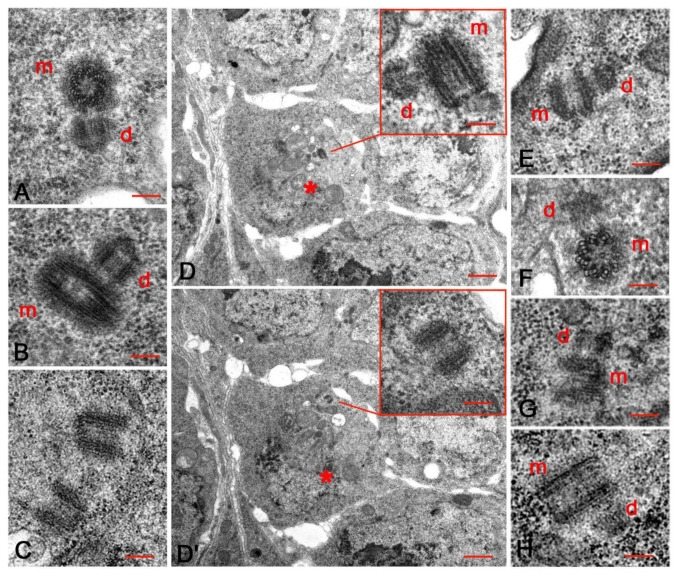
Centriole duplication during early Drosophila oogenesis. (**A**) Orthogonal parent centrioles in region 2a cystocytes. (**B**) Detail of an elongated centriole in region 2a with a short daughter. (**C**) Detail of two disengaged parent centrioles from a cystocyte in region 2a. (**D**,**D’**) Two consecutive cross sections of a group of cystocytes in region 2a from a series of four sections, above and below the focal planes represented; the cystocyte marked by the asterisk shows one elongated centriole associated with a small daughter centriole (**D**, inset) and one slightly distant, isolated centriole (inset, **D’**). Details of a pair of orthogonal centrioles within a ring canal (**E**), inside the cytoplasm of oocytes in region 2b (**F**) and stage 3 (**G**); a distinct procentriole is also visible close to one elongated centriole during stage 4 (**H**). m and d denote mother and daughter centrioles, respectively. Bars: **A**–**C**, **E**–**H**, insets **D**,**D’**,150 nm; **D**,**D’**, 1 μm.

Each cystocyte in region 2a usually held a pair of orthogonal centrioles consisting of a mother and a shorter daughter (Figure 4A,B). However, we also found parent centrioles that lost their reciprocal orientation and became slightly distant from one another (Figure 4C). Serial sections of such disengaged centrioles in region 2a often showed that one of them was associated with a small procentriole, whereas the other centriole of the same pair did not have daughters (Figure 4D,D’). The odd number of centrioles present in the same cystocyte suggests that one centriole from the same pair was unable to duplicate, or that centriole duplication of the parent centrioles occurred at different times.

Orthogonal centrioles were occasionally observed within the ring canals (Figure 4E). It is unclear if these centrioles translocated to the oocyte from the nurse cells together or if they duplicated during their travel. Orthogonal mother and daughter centrioles were also found inside the oocyte in region 2b (Figure 4F) and during later stages when the cyst cells left the germarium and entered the vitellarium (Figure 4G). Distinct daughters were also observed close to the basal end of the elongated centrioles in region 2a (Figure 4B,D), region 2b (Figure 3B,B’), and stage 4 (Figure 4H). 

By stage 2, the 15 sibling nurse cells had started the endoreplication cycle to provide the developing oocyte with all the cytoplasmic components needed for its growth, which is completed during stage 14 at the distal end of the ovariole. Serial sections of germ cell cysts during stages 2 (*n* = 11; Figure 3C) and 3 (*n* = 7; Figure 3D) showed clusters of 15–20 centrioles posterior to the oocyte nucleus and in front of the follicular epithelium. By taking serial sections of germ cell cysts during stage 4 (*n* = 4), stage 5 (*n* = 6), and stage 6 (*n* = 8), we noticed that the number of centrioles within the oocyte cytoplasm increased until stage 4, when we found 22–26 centrioles, and then decreased to 10–13 centrioles during stages 5 and 6.

### 3.3. The Centriole Structure in the Drosophila Ovariole

**Figure 5:**
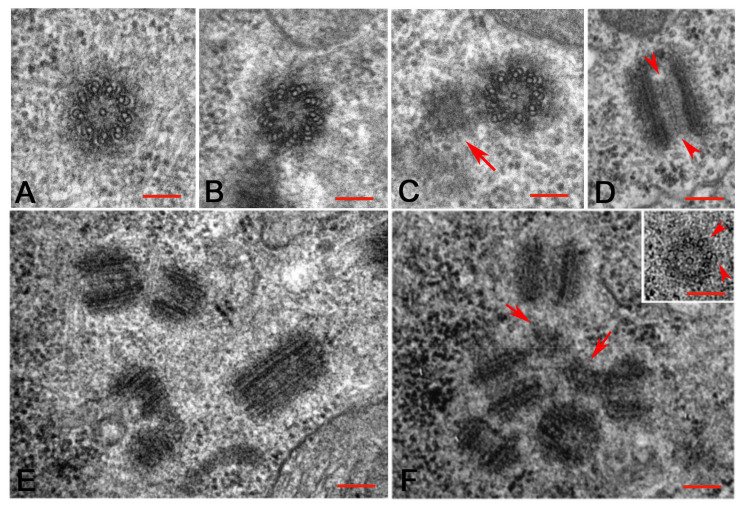
Structural details of the centrioles within the Drosophila ovariole. (**A**) Centriole from a cap cell showing the wall consisting of microtubule doublets. Details of germ cell centrioles consisting of a complete set of microtubule triplets (**B**) or mixed doublets and triplets (**C**); the centrioles with incomplete walls can support the assembly of procentrioles (arrow). (**D**) Longitudinal section of an elongated centriole showing the extension of the cartwheel (arrowheads). Details of centriole clusters in the oocyte cytoplasm during stages 4 (**E**) and 5 (**F**): note the variable length of the centrioles and the presence of centriole fragments (arrows). Short centrioles whose walls consists of single A-tubules and incomplete B-tubules (inset **F**, arrowheads) are also observed. Bars: **A**–**D**, inset **F**, 100 nm; **E**,**F**, 150 nm.

The centrioles of female GSCs and cystocytes are retained and invariably consist of nine MT triplets, whereas the centrioles of the somatic cells consist of nine MT doublets. We confirmed that the centrioles of the cap cells, escort cells, and follicle cells lack the C-tubule (Figure 5A), whereas the centrioles of the female germ cells usually consist of nine MT triplets (Figure 5B). The careful analysis of cystocytes at different developmental stages revealed that this model does not represent the only structural plane of these centrioles. The walls of 45% of the centrioles scored within the germarium (42; *n* = 93) and 58% of the centrioles scored during stages 2–5 (59; *n* = 102) consisted of mixed MT triplets and doublets (Figure 5C). This incomplete structure suggests that the centrioles did not achieve full and complete organization. However, they were able to act as mothers for procentrioles (Figure 5C). These, in turn, did not achieve the structural organization of their mothers and consisted of mixed MT singlets and doublets (Figure 3B’). We noticed that a distinct cartwheel spanned the whole length of all the centrioles, including the elongated ones (Figure 5D).

During stages 4–5 the centrioles formed compact clusters close to the oocyte nucleus. We noticed that these centrioles, not including the more elongated ones that measured 298 ± 15 nm, were highly variable in length, spanning from 125 nm to 217 nm (Figure 5E,F). Centriole fragments were also observed within these clusters (Figure 5F). Serial sections confirmed that these fragments were incomplete centrioles, not grazing sections of short oblique centrioles. Isolated short centrioles consisting of single A-tubules and incomplete B-tubules were also found during stages 3–4. The B-tubules of these centrioles were incomplete and appeared as short hook-blades (inset, Figure 5F).

## 4. Discussion

It has been reported that the Drosophila oocyte derives from one of the two older cystocytes with four ring canals [6], but the mechanisms that direct this choice are still unclear. Two models have been proposed to explain the oocyte selection: a stochastic process, in which the choice of the oocyte occurs randomly late in region 2a, and a predetermined mode, in which the cystoblast inherits an intrinsic asymmetry just before the first mitosis [3]. Although not completely exhaustive, the second hypothesis appears to be the most likely. The asymmetric distribution of the fusome material suggests that the future oocyte is already selected at the first mitotic division and could be identified as the cystoblast [18]. Thus, the only sign of early asymmetry is that one of the two pro-oocytes with four ring canals maintains a fusome portion derived from the spectrosome and inherited after the asymmetric division of the stem cell. Remarkably, the fusome seems to have a main role in the polarization of the cytoskeletal elements needed to deliver selected information to one of the two pro-oocytes [2]. After the formation of the 16-cell cysts, however, this early asymmetry is lost and can no longer be detected. 

**Figure 6:**
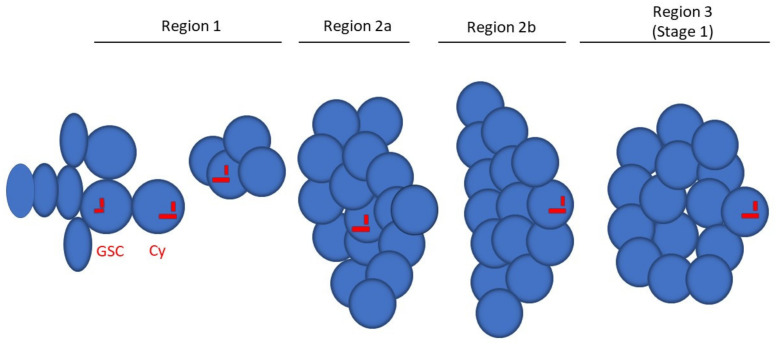
Schematic drawing of the early stages of oogenesis in the Drosophila germarium depicting the localization of the elongated centriole (GSC, germline stem cell; Cy, cystoblast).

Here, we report a new distinctive sign of asymmetry that could help to identify the oocyte immediately after the division of the germline stem cell. Our ultrastructural analysis of the Drosophila germarium allowed us to identify a centriole which was much longer than the other centrioles in the germ cell cysts. Serial and random sections of several 16-cell cysts showed that this elongated centriole was present in only one cell from each cyst. Remarkably, this unique centriole was always associated in region 2a with one of the two pro-oocytes, and it was found in region 2b in the cytoplasm of the posterior cell that retains the synaptonemal complex and accumulates multiple centrioles (Figure 6). These observations led us to infer that this unique centriole present in the cystoblast is directly inherited by the oocyte, thus representing a clear asymmetry which is established just after the division of the stem cell.

The elongated centriole displays a cartwheel that extends for the entire length of its wall. This aspect contrasts with the architecture of the centrioles found during Drosophila spermatogenesis. Male GSCs, spermatogones and young primary spermatocytes, display a cartwheel that extends the full length of the short centrioles. The cartwheel remains constant in size whilst the dimensions of the centrioles increase by up to 8–10 times during the prophase of the first meiosis [19]. Therefore, a mechanism that maintains the length of the cartwheel seems to be present during Drosophila male gametogenesis. The elongated centriole found in the Drosophila oocyte does not possess, nor does it escape, the controls that keep the cartwheel at a constant size. If this is so, why is this condition not inherited by the cell’s progeny, which instead show short centrioles?

The finding that, during late telophase, GSCs display one pole with a long/short centriole pair and another pole with a short/short pair suggests that this centriole asymmetry could be already present during the previous interphase. It remains unclear why the elongated centriole was inherited at telophase by the cystoblast, rather than staying in the GSC. Loss of function of asterless (asl), a core centriolar component [20], and Klp10A, a microtubule-depolymerizing kinesin of the kinesin-13 family, [21,22] results in mother centriole-specific elongation in Drosophila male GSCs, suggesting that this centriole has distinct and unique microtubule dynamics. We can hypothesize that the mother centriole of the female germ stem cells also has an inherent tendency to elongate, but without the control mechanisms of the male germ line. In Drosophila, the asymmetric germ stem cell division is accompanied by a stereotypical inheritance of the parent centrioles; the mother centrosome is consistently inherited by the male stem cells [23], whereas the female stem cells retain the daughter centrosome [24]. We suggest that the elongated mother centriole of the female stem cell might be inherited by the cystoblast and maintained during the further mitotic divisions, in association with the remnant spectrosome of the differentiating oocyte. Remarkably, the persistence in the oocyte cytoplasm of a mother centriole inherited after the asymmetric division of the germline stem cells was postulated several years ago in a visionary cartoon [25]. It is unclear if the elongated centriole has specific functions in oocyte development, and the function of all the centrioles during the early Drosophila oogenesis remains elusive. The main conundrum is the observation that Sas4 mutant flies lack centrioles but lay eggs [26,27], suggesting that centrioles are dispensable for oocyte development in Drosophila.

We noticed during our ultrastructural analysis that distinct procentrioles are not restricted to the dividing stem cells and cystocytes but are also present in regions 2a and 2b when the 16 germ cells stop dividing. These observations confirm and extend previous reports [12,14] showing that post-mitotic centrioles can duplicate in the 16-cell cysts of region 2a, and that this happens independently of the DNA replication that ceases after the fourth mitotic division. This is an intriguing point because canonical centriole duplication is tightly coupled with DNA replication to ensure the correct number of centrosomes during each cell cycle [28]. Post-mitotic centrioles of hub cells [19], somatic cyst cells [29], and ommatidia [30] are unable to duplicate, suggesting the presence of mechanisms which seek to avoid unnecessary replication in these tissues. Presumably, the centrioles of the Drosophila germarium escape or turn off these controls. This diverges in female and male gametogenesis because the spermatocyte centrioles do not duplicate in the absence of DNA synthesis. Therefore, the centrioles of the male and female germ cells seem to be subject to different rules and answer to different requirements. Remarkably, we also observed orthogonal centrioles during stages 3 and 4.

The presence of odd centriole numbers within the same cells in region 2a, before the process of centriole migration, suggests that the duplication of the parent centrioles is an asynchronous process that overrides the usual cell-cycle controls. The possibility that centrioles replicate in region 2a and maintain their close association until stages 3 and 4 is hard to believe. This observation points to a later duplication process, presumably within the vitellarium. Accordingly, the variable length of the centrioles during these stages could be the result of replication events occurring at different times.

No structural differences between the post-mitotic centrioles of the female germ cells have been reported until now. Most of the germ cell centrioles found within the germarium and the early stages of the vitellarium display a slight variation in length and an incomplete tubular wall, often consisting of mixed MT doublets and triplets. Therefore, these centrioles cannot reach maturity with a complete set of triplet MTs. They can duplicate, however, suggesting that, at least in the Drosophila germarium, centriole replication does not require triplets. Functionally active somatic and early embryonic centrioles consist of nine doublets [31]. Therefore, nine doublets is the minimum requirement for a proper duplication, and the presence of C-tubules is irrelevant. If this is the case, what is the meaning of the C-tubule? Why is this tubule only present in the germline? A simple explanation is that the C-tubule is needed during Drosophila spermiogenesis to assemble the accessory fibres of the sperm axoneme [32], but this function is unnecessary during oogenesis.

Although, the centrioles appear to be useless organelles during oogenesis, they must be eliminated because their retention is deleterious for embryonic development. Thus, the mature oocyte lacks centrosomes [33]. Centriole elimination during oogenesis is needed to ensure the correct centriole number upon fertilization, and it is also thought to prevent parthenogenetic development. The ectopic expression of the kinase Polo, the major PCM recruitment factor, prevents PCM loss and leads to functional maternal centrosome retention at the end of oogenesis [14]. How centriole disassembly occurs is still unclear. The discovery of very short procentrioles consisting of A-tubules and incomplete B-tubules in the oocyte cytoplasm of regions 3 and 4 could be explained through a tardive replication process and a consequent delay in growth. However, the procentrioles are not associated with mothers and are isolated in the cytoplasm. This poses the question of how these incomplete centrioles can maintain their stability during oogenesis and suggests that the reduced wall with incomplete B-tubules could represent the first morphological sign of centriole disassembly. Thus, the centriole disassembly could start very early in the vitellarium. Accordingly, we noticed a consistent reduction in centriole numbers in the oocyte cytoplasm during stages 5 and 6.
